# Acute Unilateral Renal Infarction in the Setting of an Inherited Thrombophilia and Atrial Septal Defect

**DOI:** 10.1155/2017/3159363

**Published:** 2017-08-27

**Authors:** Siavash Piran, Sam Schulman

**Affiliations:** Department of Medicine, Division of Hematology and Thromboembolism and Thrombosis and Atherosclerosis Research Institute, McMaster University, Hamilton, ON, Canada

## Abstract

We present a case of renal infarction in a 43-year-old female with history of stroke at age 14. She was found to be heterozygous for the prothrombin G20210A gene mutation. Loop monitoring revealed no atrial fibrillation. Transthoracic and transesophageal echocardiograms showed no thrombus. However, there was a small shunt due to an atrial septal defect (ASD). She was treated with warfarin and had device closure of her ASD. This was a suspected case of paradoxical embolism through an ASD leading to renal infarction.

## 1. Introduction

Paradoxical embolism is a rare but increasingly recognized cause of embolic events. An atrial septal abnormality such as a patent foramen ovale (PFO) or an atrial septal defect (ASD) serves as a pathway for a thrombus from the peripheral veins, bypassing the lungs, and entering the systemic circulation [1]. Cryptogenic stroke is the most commonly described presentation in patients with paradoxical embolism [2]. Renal infarction secondary to paradoxical embolism has rarely been described. Here, we report a case of a paradoxical embolism caused by ASD involving only one kidney in the setting of an inherited thrombophilia.

## 2. Case Presentation

A 43-year-old female was seen in consultation at our thrombosis clinic. She had a stroke at age 14 and had presented with collapse and left sided hemiparesis. Her thrombophilia work-up was positive for a prothrombin G20210A gene mutation in heterozygous form. She had been on aspirin 81 mg daily since age 14.

Prior to being diagnosed with a renal infarct at age 42, the patient presented with nausea, vomiting, hematuria, and left flank pain and was initially diagnosed as renal colic. She subsequently had a computerized tomography scan of the abdomen and pelvis, which showed evidence of a wedge-shaped area in the lower pole of the left kidney consistent with a renal infarction. She was not on an oral contraceptive. We started treatment with intravenous heparin and transitioned to warfarin for 15 months without any further thromboembolic events.

Given that cardioembolic sources are well-documented causes of renal infarction [3], the patient had loop monitoring for two weeks and electrocardiograms, which did not detect atrial fibrillation. She also had two echocardiograms, none of which showed any evidence of cardiac thrombus. A transthoracic echocardiogram was performed with agitated saline at rest and after valsalva maneuver, which showed mild to moderate degree of shunting at rest that increased significantly with the release phase of a valsalva maneuver. This was suspicious for a PFO. A follow-up transesophageal echocardiogram showed a small left to right shunt due to a small ASD rather than a PFO. The patient had device closure of the ASD with no evidence of any remaining shunt on a transthoracic echocardiogram. It is likely that the patient's renal infarction was related to paradoxical embolism caused by small deep vein thrombosis migrating through the ASD shunt.

After 4 months of being off of anticoagulation, patient had a D-dimer test, which was positive at 591 *μ*g/L. There were no other reasons for the elevated D-dimer. Based on an annual risk of recurrence of approximately ten percent in females with a first unprovoked venous thromboembolism (VTE) event and a positive D-dimer, the patient was restarted back of warfarin [4].

## 3. Discussion

Prothrombin gene mutation is the second most common inherited thrombophilia with a prevalence of approximately 2% [5]. The risk of VTE in individuals who are heterozygous for the prothrombin G20210A mutation is approximately 3-4-fold compared with a control group [6, 7]. It is unclear if the prothrombin gene mutation increases the risk of VTE recurrence, with some studies suggesting an increased risk [8] while others not [9, 10]. It is generally known that the prothrombin gene mutation is not associated with an increased risk of arterial thrombosis. However, a 2017 meta-analysis reported a slightly increased risk of stroke in children and young adults with the prothrombin gene mutation [11].

There are several case reports of paradoxical embolism causing renal infarction through a PFO in presence or absence of a thrombophilia (Table 1) [12–17]. However, to our knowledge, this is the first report of a renal infarction due to paradoxical embolism in the setting of an ASD and thrombophilia. Paradoxical embolism is a rare cause of renal infarction; however the role of an atrial septal abnormality as a source of embolic events in various organs is increasingly recognized.

## 4. Conclusions

Our case report identifies paradoxical embolism causing renal infarction through an ASD and highlights the need for immediate identification of a paradoxical embolism so that anticoagulation can be started and device closure can be considered to prevent further embolic events in other organs.

## Figures and Tables

**Table 1:** Case reports of renal infarction associated with paradoxical embolism in the setting of a patent foramen ovale.

Study	Garachemani et al.	Iwasaki et al.	Jeong et al.	Ekinci et al.	Vilbert and Franciosa	Khoma et al.
Year of publication [Ref]	2007 [12]	2011 [13]	2012 [14]	2014 [15]	2016 [16]	2016 [17]

Renal (unilateral versus bilateral, side)	Unilateral, left side	Unilateral, right side	Unilateral, right side	Unilateral, left side	Unilateral, left side	Unilateral, left side

Venous thromboembolism detected	No investigations performed	No DVTs detected	No DVTs or PE detected	No investigations performed	No DVTs detected	No DVTs detected

Thrombophilia	No investigations performed	Negative screen	Negative screen	Negative screen	Prothrombin G20210A mutation	Negative screen

Other VTE risk factors	None	None	None	None	OCP	After bariatric surgery

Other organ involvement	Myocardial infarction	None	None	None	None	None

Anticoagulation	Oral anticoagulation type and duration unspecified	Secondary prevention with aspirin 100 mg daily	IV heparin	Enoxaparin 60 mg twice daily	IV heparin transitioned to warfarin, 6-month duration	Warfarin, 6-month duration

Device closure	Yes	Not specified	Planned	Not specified	Not planned	Planned

DVT: deep vein thrombosis; IV: intravenous; OCP: oral contraceptive pill; PE: pulmonary embolism.
